# Machine learning-based assessment of condylar changes after orthognathic surgery of asymmetric dentofacial deformities

**DOI:** 10.3389/fsurg.2025.1594849

**Published:** 2025-09-18

**Authors:** Jin Tang, Jiayu Shi, Shuguang Liu

**Affiliations:** Stomatological Hospital, School of Stomatology, Southern Medical University, Guangzhou, Guangdong, China

**Keywords:** orthognathic surgery, asymmetry, condyle, automated methods, temporomandibular joint

## Abstract

**Introduction:**

This retrospective study aimed to investigate three-dimensional (3D) condyle change including volumetric, positional, rotational alterations in patients with asymmetric dentofacial deformities immediate and long after bimaxillary orthognathic surgery.

**Methods:**

The sample included 36 patients who underwent bimaxillary orthognathic surgery, with a maxillomandibular dental midline deviation >3 mm, excluding those with class II/III malocclusions and craniofacial syndrome. A fully automated deep learning-based assessment method was used to analyze the volume, position and rotation of condyle based on Cone-beam Computed Tomography (CBCT) images. Repeated measures ANOVA was used to compared the changes at three intervals—pre-surgery (T0), one-week post-surgery (T1), and six months post-surgery (T2)—of the deviated sides and non-deviated sides condyle.

**Results:**

The condyle on the deviated side was smaller than that on the non-deviated side, with significant volume reductions observed six months post-surgery on the deviated side. Several condylar changes were observed immediately after surgery, though of small magnitude, and it mostly tended to return to their original positions 6 months after surgery. However, the condyle rotated laterally on the deviated side and medially on the non-deviated side post operation and in long-term.

**Conclusion:**

For patients with asymmetry, condyle on the deviated side undergo greater remodeling than the non-deviated side after orthognathic surgery. There are measurable rotations in the coronal plane of condyle on both sides.

## Introduction

Asymmetric dentofacial deformity frequently results in aesthetic and functional impairments, typically characterized by pronounced jaw deviation, imbalance, and occlusal disorders (1–4). The causes of asymmetric dentofacial deformities are primarily associated with genetic factors, developmental abnormalities, and trauma (1, 5). Additionally, studies have identified pathological changes in the temporomandibular joint (TMJ), such as excessive load or positional deviations of the condyle, as significant contributors to jaw asymmetry (1). Asymmetric joint movement may result in either overgrowth or undergrowth of the condyle, subsequently contributing to facial deformity (6). Currently, the primary treatment for patients with severe skeletal asymmetry involves orthodontic treatment combined with orthognathic surgery (7). The occlusal plane will be adjusted through maxillary Lefort I osteotomy. After bilateral sagittal split mandibular ramus osteotomy (BSSRO), asymmetric movement of the distal mandibular segment facilitate the adjustment of the mandible's length, height, and width on both sides. The jaws will be repositioned to restore facial aesthetics and improve occlusion (1, 8). However, due to the variability in condylar position during surgery, changes in condyle after orthognathic surgery are highly intricate, affecting the long-term outcomes of the surgery (1, 9, 10).

Advancements in imaging technology have led to significant changes in the methods used to analyze TMJ alterations (11, 12). The Cone-Beam Computed Tomography (CBCT) enhanced the accuracy of condylar position assessment, overcoming the limitations of two-dimensional imaging, and become widely utilized in orthognathic surgery research (13–15). Nowadays, machine learning-based assessment emerged to analyze the image data automatically and effectively. Our previous research has developed a fully automated quantitative method using deep learning, capable of identifying the condyle in CBCT images and measuring changes in its position and volume before and after surgery. This method enables a more objective and accurate evaluation of postoperative condylar changes in patients with skeletal malocclusion, providing a scientific foundation for assessing surgical outcomes and clinical treatments (16). In the previous study, we found that condylar resorption in both sides after bimaxillary orthognathic surgery, correlated with counterclockwise rotation in the sagittal plane in patients with skeletal class II malocclusion. In the current study, we used a new sample with asymmetric dentofacial deformity.

Currently, studies on condylar changes in patients with Angle class II and class III jaw deformities have revealed several similar trends (17). In Angle class II patients, the condyle often exhibits significant posterior displacement and remodeling due to the anterior displacement of the distal mandibular segment (14, 18, 19). In contrast, patients with Angle Class III, who present with protruding mandibles, typically require resection of part of the proximal segment and posterior displacement of the distal segment. Although these patients undergo some remodeling and movement of the condyle, such changes have a limited impact on bone recurrence and clinical outcomes (13, 14). However, research on condylar changes in patients with asymmetric dentofacial deformities remains limited (1, 11, 12). Systematic studies evaluating condylar changes before and after surgery, as well as the long-term effects of these changes on joint function, are lacking in this patient population.

This study aimed to measure condylar volume, position, direction in patients with asymmetric dentofacial deformities before and after surgery using an established automated measurement program. The hypothesis was that condylar kinematics and spatial orientation would be significantly different between the deviated and non-deviated sides from pre-surgery to 1-week and 6-month post-surgery.

## Materials and methods

### Study design and patient selection

This study was approved by the Ethics Committee of the Stomatological Hospital of Southern Medical University [Approval No. NYKQ-EC- (2024)08] and all participants provided informed consent. This study included patients with asymmetric dentofacial deformities who underwent bimaxillary orthognathic surgery between 2020 and 2023 in the Southern Medical University Stomatological Hospital for retrospective analysis.

Inclusion criteria include:

a. patients diagnosed with asymmetric dentofacial deformity, with a maxillomandibular dental midline deviation >3 mm and a skeletal Class I relationship. The molar relationship was Class II on the deviated side and Class III on the non-deviated side.
b. patients underwent bimaxillary orthognathic surgery including LeFort I osteotomy and BSSRO
c. the CT scan images at three intervals were available

Exclusion criteria include:

a. patients associated with class II or class III malocclusions
b. patients with a history of craniofacial syndrome, craniomaxillofacial trauma or surgery
c. patients exhibiting obvious symptoms of temporomandibular joint dysfunction

**Figure 1:**
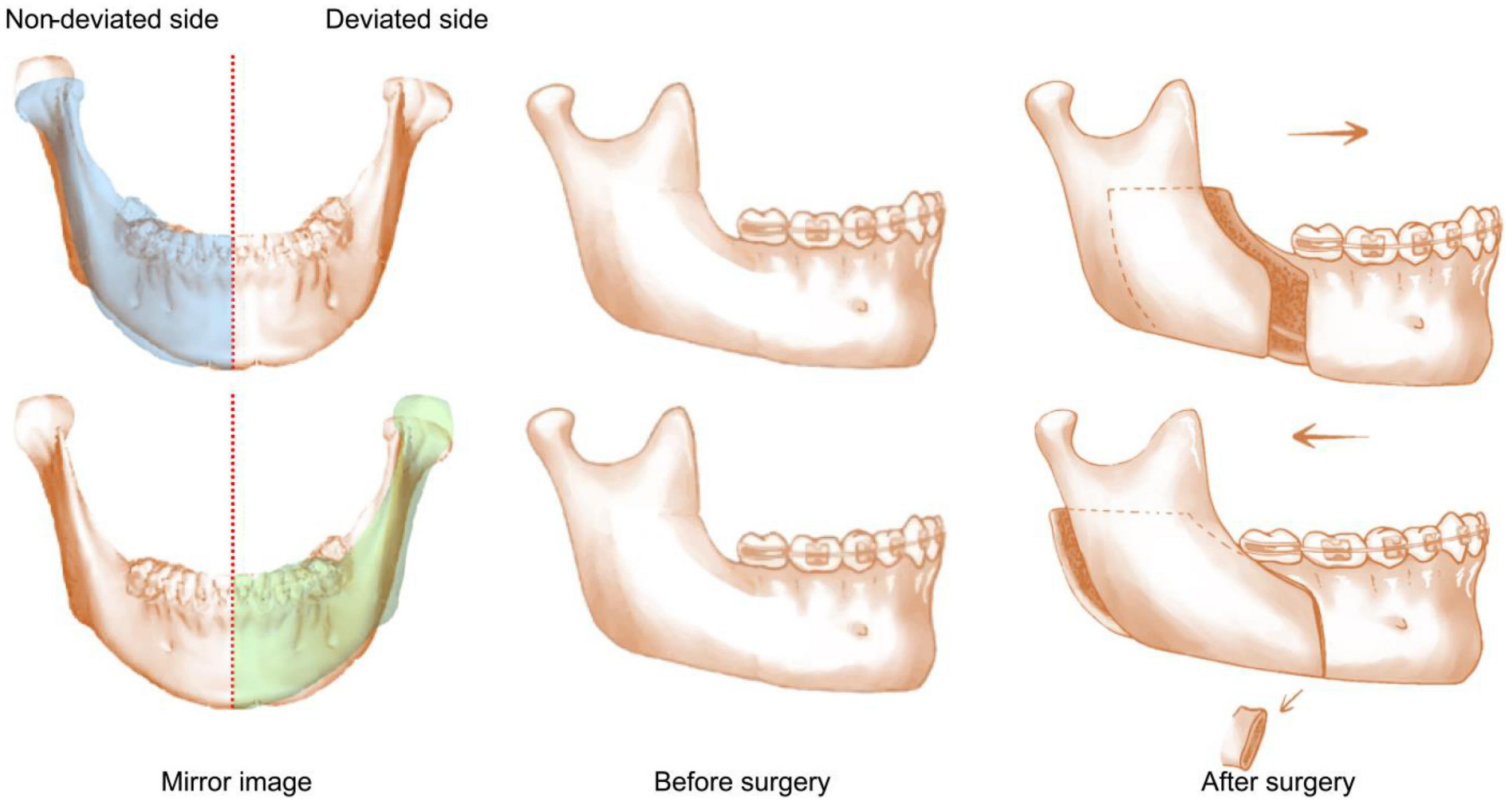
The condyle on the side of chin deviation is defined as the deviated condyle, while the contralateral condyle is defined as the ninon-deviated condyle. The red line refers to the midline of face. The blue area is the mirror image of deviated side and the green area is the mirror image of non-deviated side. On the deviated side, the distal mandibular segment moves forward. On the non-deviated side, the distal mandibular segment moves backward and thus the proximal mandibular segment should be cut.

We found 122 patients with dentofacial deformity and 30% of them had asymmetry whose skeletal and dental midline between maxillary and mandible deviated >3 mm. 86 patients were excluded according to the criteria. All patients received conventional orthodontic treatment before surgery, and post-surgery orthodontic adjustments were scheduled to begin 3 weeks after surgery. For these reason, patients' dental midline and skeletal midline were mostly aligned. Orthognathic surgery was performed by three experienced maxillofacial surgeons with over 15 years of experience. All surgeries were digitally planned. The condyle on the side of chin deviation was defined as the deviated condyle, while the condyle on the other side of chin deviation was defined as the non-deviated condyle. During the procedure, the jaw position was accurately determined using the bite guide. Since we excluded the patients associated class II or class III malocclusions so that during the operation, the distal mandibular segment on the deviated side was moved forward while the other side was move backward (Figure 1). The fixed position of the bone segments was confirmed using the bite guide. Besides, the sample did not include individuals with obstructive sleep apnoea.

### Radiographic evaluation

CBCT (New Tom VG, Via Silvestrini, 20 37135 Verona, Italy) was used to collect raw data in DICOM format. The CBCT scanning parameters included 110k Vp, voxel size of 0.3 mm × 0.3 mm × 0.3 mm, and a field of view of 150 mm × 150 mm. The single scan time was 15 s with 360° rotation acquisition. All patients' CBCT data were acquired using the same machine under consistent conditions. During the scan, the patients sat upright with their eyes facing forward, lips naturally relaxed, Frankfort Horizontal planes parallel to the ground, and molars in the intercusp position.

All CBCT data were anonymized following collection and subsequently analyzed. Each patient's CBCT scans were taken at three time points: within 1 month prior to surgery (T0), approximately 1 week after surgery (T1) and between 6 months post-surgery (T2). All CBCT data were anonymized following collection for subsequent analysis and examiners were blinded to measure the data of different times.

### Measurement of condylar displacement, rotation, and volume

A fully automated quantitative method was developed based on previous studies to segment and measure the condyle in the anonymized CBCT images (16). The area from the horizontal line of the lowest point of the sigmoid notch to the mandibular lingula was defined as the surgical stable region. The stable region from the cropped T1, T2 area was then aligned onto T0 for fine registration. Upon aligning the condyles across T0, T1, and T2, we obtained transformation matrices for any two coordinate sets. Changes of condyles were assessed by applying these matrices.

**Figure 2:**
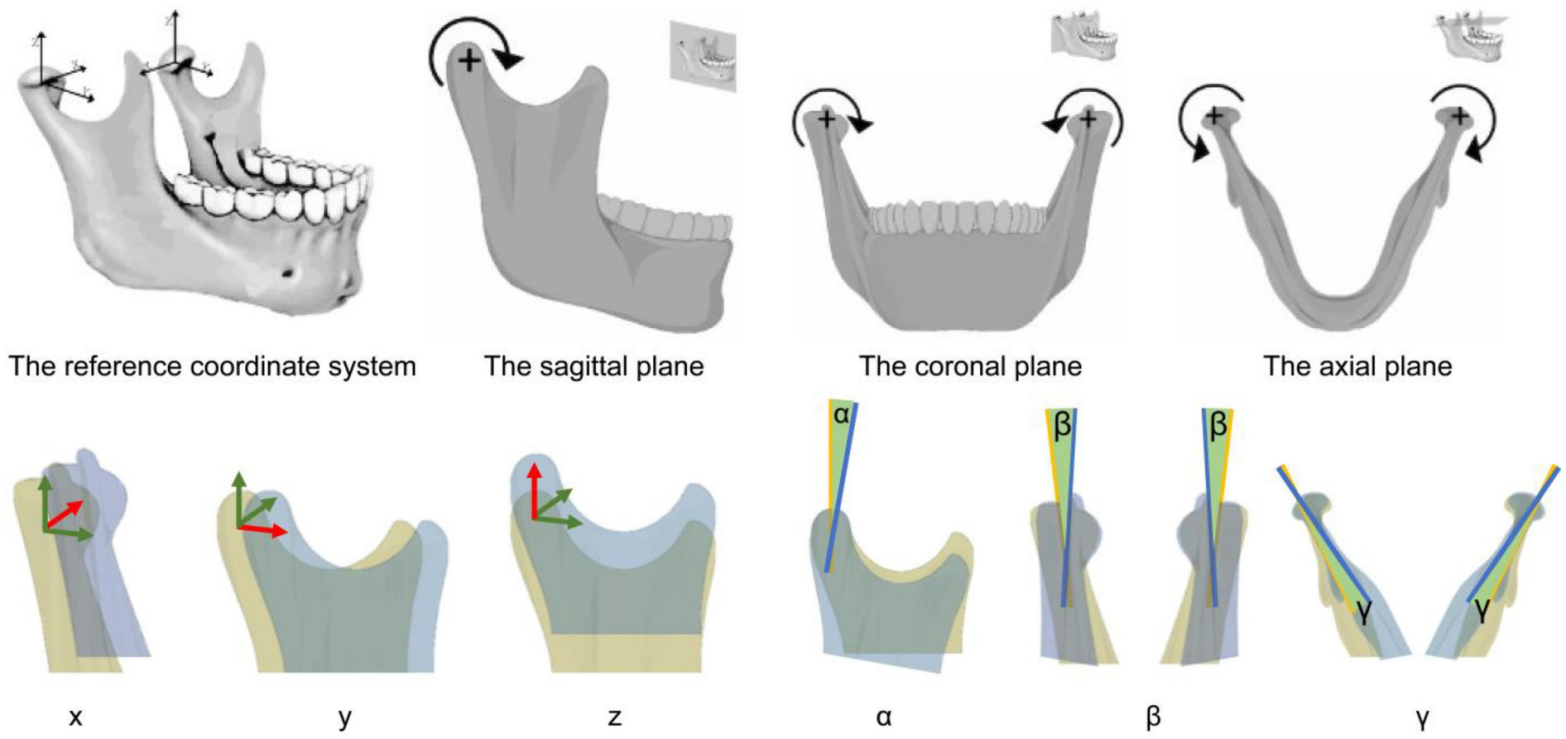
The direction of the movement parameters and rotation parameters. The red arrows show the direction in the reference coordinate system. The yellow area is the position of condyle before surgery whereas the blue area is the position of condyle after surgery. The green area shows are rotation angle of condyle.

The reference coordinate system was defined with the image center as the origin. The X, Y, and Z axis, and the sagittal plane (pitch), coronal plane (row), and axial plane (yaw), are oriented accordingly (Table 1; Figure 2). Subsequently, we cropped the aligned mandible at T0, T1, and T2 to assess the condylar volume, utilizing the lowest point of the sigmoid notch identified in T0.

**Table 1:** The definitions of the parameters.

Parameter	Definition	
X	The distance of the lateral movement of condyle	
	+ medial movement toward the midline	− movement against the midline
Y	The distance of the anterior-posterior movement of condyle	
	+ forward movement	− backward movement
Z	The distance of the vertical movement of condyle	
	+ upward movement	− downward movement.
*α*	The rotation angle of condyle in the sagittal plane along the *X* axis	
	+ pitch down	− rotate anticlockwise in the sagittal plane
*β*	The rotation angle of condyle in the coronal plane along the *Y* axis	
	+ condyle medially rotate	− condyle laterally rotate
*γ*	The rotation angle of condyle in the axial plane along the Z axis	
	+ anterior-medial rotation	− posterior-lateral rotation

### Statistical analysis

Statistical analyses were conducted using SPSS version 26.0. Initially, all quantitative variables were assessed for normality using the Shapiro–Wilk test. Normally distributed variables were expressed as mean ± standard deviation (SD). Repeated measures ANOVA were applied to assess the statistical difference of condylar volume, translational changes (X, Y, Z axes) and rotational displacements (pitch, roll, yaw) at T0, T1, and T2 for each side. Side and time were treated as variables with time as a repeated measure. And Dunnett *post hoc* tests were used with corrections if appropriate. A statistical significance threshold of *α* = 0.05 was applied for two-tailed tests.

## Results

### Basic characteristics of the subjects

Out of 36 patients comprised 8 males (22.2%) and 29 females (77.8%), with a mean age of 23.0 years and a mean ANB angle of 0.32°. Chin deviation was left-sided in 15 patients and right-sided in 21 patients. A total of 36 pairs of condyles were stratified into deviated and non-deviated subgroups based on chin deviation direction. There were not sample of patients experiencing TMJ discomfort, and there was no patient underwent a surgery only procedure and with adequate dental occlusion. Shapiro–Wilk test showed that the data conformed to normal distribution (*p* > 0.05).

### Changes in condylar volume before and after surgery

**Figure 3:**
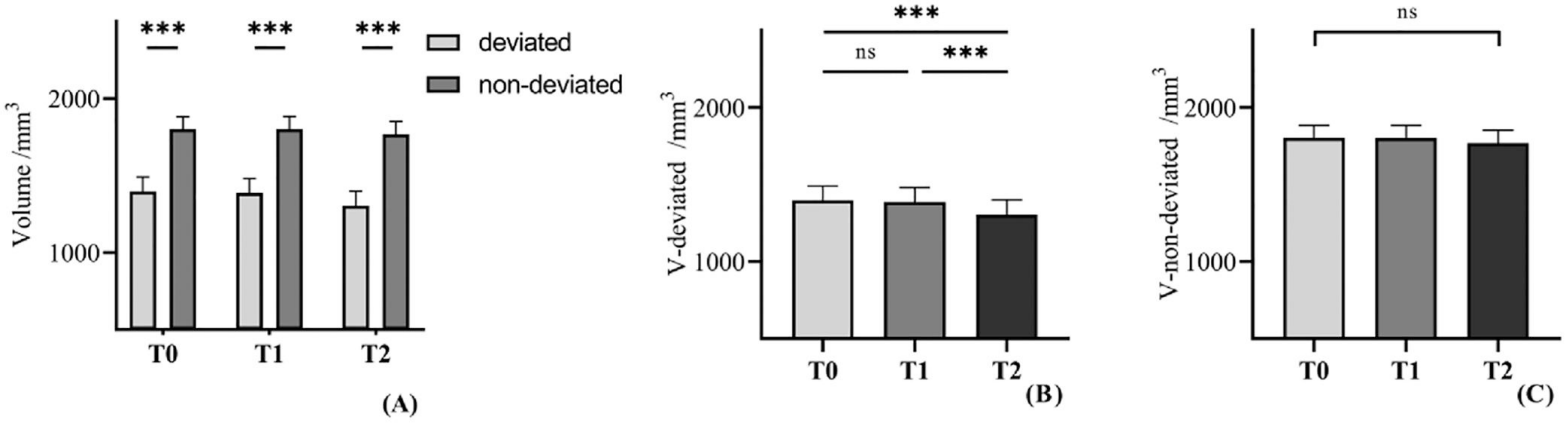
**(A)** Comparison of condylar volume between the deviated side and the non-devaited side. **(B,C)** Comparison of condylar volume between diverse stages in each side. Repeated measures ANOVA were used to evaluate the significance of main effect and *post-hoc* pairwise comparisons with Bonferroni correction. Ns, significance. ***: *p* < 0.001.

The mean value of the condylar volume on the deviated side was 1,396.6 cm³ ± 559.5 cm³, while the volume of the condylar volume on the non-deviated side was 1,802.5 cm³ ± 489.0 cm³ (Table 2). The condylar volume on the deviated side was significantly smaller than the non-deviated sides (*p* < 0.001) (Figure 3).

**Table 2:** Comparison of condylar volume between diverse stages, using repeated measures ANOVA.

Condyle	T0 (mm^3)^ Mean ± SD	T1 (mm^3)^ Mean ± SD	T2 (mm^3)^ Mean ± SD	Significance		
				F	*P-*value	*η* ^2^
Deviated side	1,396.6 ± 559.5	1,386.6 ± 553.2	1,303.3 ± 576.0	17.5	<0.001***	0.33
Non-deviated side	1,802.5 ± 489.0	1,800.8 ± 500.5	1,767.0 ± 515.9	3.1	0.071	0.08

T0, before surgery; T1, 1-week after surgery; T2, more than 6-months after surgery; SD, standard deviation.

*** *p* < 0.001. Partial eta-squared (*η*^2^): the effect size of factors on independent variables. 0.01: small effect size; 0.06: medium effect size; 0.14: large effect size.

The volume of the condyle on the deviated side was significantly difference (*p* < 0.001) (Table 2). Using *post hoc* tests, the change of the condylar volume from T1 to T0 was not statistically significant (*p* = 0.762), whereas the volume of the condyle six months after surgery was significantly smaller compared with one week after surgery (T2 to T1) (*p* < 0.001).

The average volume of the condyle on non-deviated side was shown in Table 2. In contrast, repeated measures ANOVA showed no significant changes of condylar volume among three times (*p* = 0.071), suggesting that the condyle volume on non-deviated side remained stable.

### Position changes of condyle before and after surgery

**Figure 4:**
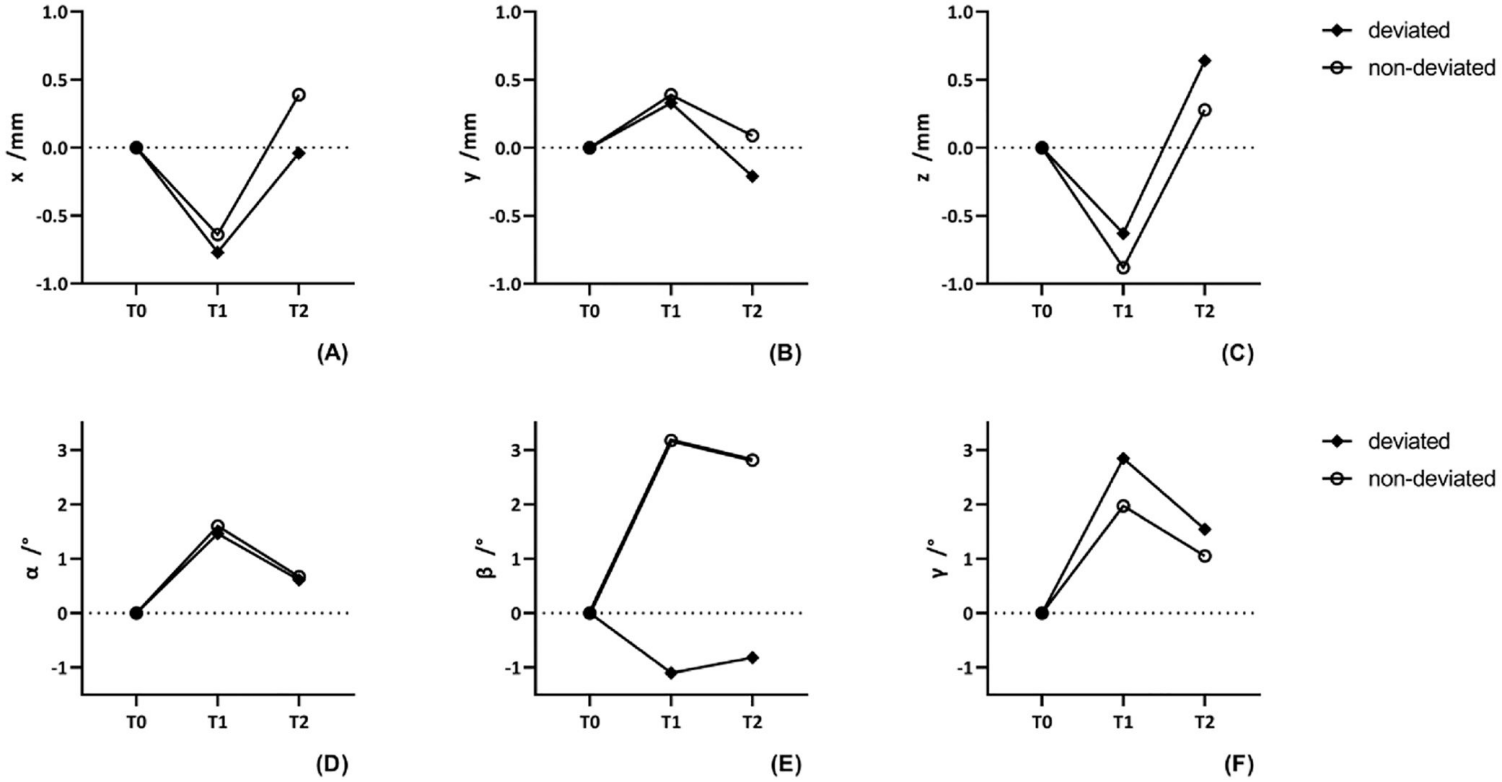
The currency of position and rotation change of condyle in different side at three times. **(A–C)** The positional change of condyle in different axis. **(D–F)** The rotational change of condyle in different plane.

**Figure 5:**
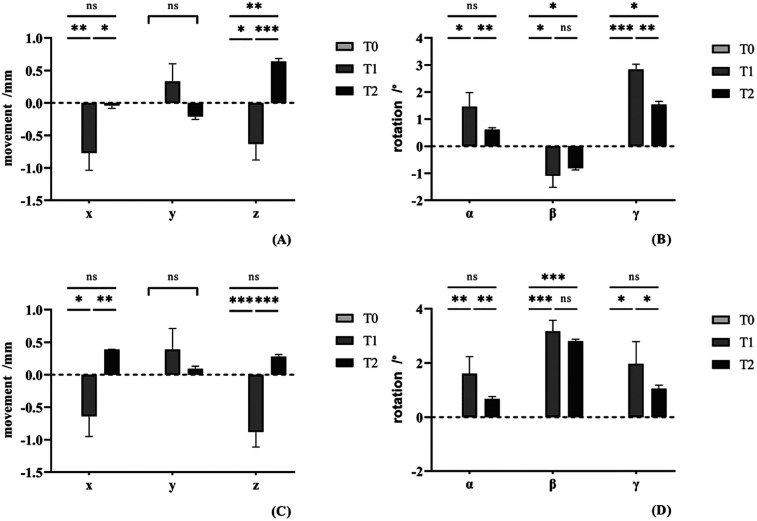
*Post-hoc* pairwise comparisons of the position and rotation change of condyle in different side at three times. **(A,B)** The positional and rotational change of condyle in the deviated side. **(C,D)** The positional and rotational change of condyle in the non-deviated side. ns, no significance. *p < 0.05. **p < 0.01. ***p < 0.001.

The position changes of condyle among three times were seen in Table 3. Significant changes were noted in the lateral movement and downward movement among different time and there no significant difference between each side. Using *post hoc* tests, in the lateral movement, condyle on the deviated side moved laterally at T1 (*p* = 0.009) and moved medially between T1T2 (*p* = 0.024). There was no significance between T2 and T0 (*p* = 0.877). Similarly, Condyle on the non-deviated side move laterally at T1 (*p* = 0.030) and medially between T1T2 (*p* = 0.002). No significant difference between T2 and T0 (*p* = 0.137). In the vertical direction, the deviated side condyle moved downward at T1 (*p* = 0.011) and moved upward between T1T2 (*p* < 0.001). Significant change was found between T2 and T0 (*p* = 0.007). The non-deviated side condyle moved downward at T1 (*p* < 0.001) and upward between T1T2 (*p* < 0.001), but there no significance between T2 and T0 (*p* = 0.231) (Figures 4, 5).

**Table 3:** The change of condylar position among three times, using repeated measures ANOVA.

Parameter		Mean ± SD (mm)Mean ± SD (mm)		ANOVA		
		T1	T2	F	*P*-value	*η* ^2^
X	Deviated	−0.8 ± 1.6	0.0 ± 0.3			
	Non-deviated	−0.6 ± 1.9	0.4 ± 0.2			
	Time			10.4	<0.0001****	0.13
	Group			0.8	0.380	0.01
	Time×group			0.6	0.556	0.01
Y	Deviated	0.3 ± 1.6	−0.2 ± 0.3			
	Non-deviated	0.4 ± 1.9	0.1 ± 0.2			
	Time			1.9	0.149	0.03
	Group			0.5	0.498	0.01
	Time×group			0.2	0.727	<0.01
Z	Deviated	−0.6 ± 1.5	0.6 ± 0.3			
	Non-deviated	−0.9 ± 1.4	0.3 ± 0.2			
	Time			25.5	<0.0001****	0.27
	Group			1.2	0.280	0.02
	Time×group			0.6	0.561	0.01

SD, standard deviation; T1, 1-week after surgery; T2, more than 6-months after surgery; X, the distance of lateral movement; Y: the distance of anterior-posterior movement; Z, the distance of vertical movement.

**** *p* < 0.0001. Partial eta-squared (*η*^2^): the effect size of factors on independent variables. 0.01: small effect size; 0.06: medium effect size; 0.14: large effect size.

### Rotation changes of condyle before and after surgery

In the measurements of the condylar rotation, significant changes were noted in all the measurements regarding pitch, roll, and yaw among different time (Table 4). In the coronal plane, main effect between different side and interaction effect was significance as well. The condyle on the deviated side rotated laterally at T1 (*p* = 0.010), whereas the non-deviated side rotated medially (*p* < 0.001). There was no considerable difference between T2 and T1 in both sides. In the sagittal plane, condyle rotated clockwise at T1 (*p* = 0.014) and then counterclockwise between T1T2 (*p* = 0.002) on the deviated side. Condyle in the other side rotated similarly. And there no significant difference between T2 and T0. In the axial plane, condyle rotated anterior at T1 (*p* < 0.001) and then rotated posterior between T1T2 (*p* = 0.003) on the deviated side. Condyle on the non-deviated side rotated anterior at T1 (*p* = 0.011) and then rotated posterior between T1T2 (*p* = 0.032). Between T2 and T0, there was significant difference on the deviated side (*p* = 0.037), but there was no on the non-deviated side (*p* = 0.152) (Figures 4, 5).

**Table 4:** The change of condylar rotation among three times, using repeated measures ANOVA.

Parameter		Mean ± SD (degree)		ANOVA		
		T1	T2	F	*P*-value	*η* ^2^
α	Deviated	1.5 ± 3.1	0.6 ± 0.4			
	Non-deviated	1.6 ± 3.8	0.7 ± 0.5			
	Time			11.1	<0.0001****	0.14
	Group			<0.1	0.896	<0.01
	Time×group			<0.1	0.931	<0.01
β	Deviated	−1.1 ± 2.5	−0.8 ± 0.4			
	Non-deviated	3.2 ± 2.4	2.8 ± 0.4			
	Time			10.0	<0.001***	0.13
	Group			56.3	<0.0001****	0.4
	Time×group			38.6	<0.0001****	0.4
γ	Deviated	2.8 ± 1.1	1.5 ± 0.7			
	Non-deviated	2.0 ± 5.0	1.1 ± 0.8			
	Time			13.6	<0.0001****	0.16
	Group			0.5	0.503	0.01
	Time×group			0.4	0.510	0.01

SD, standard deviation; T1, 1-week after surgery; T2, more than 6-months after surgery; α, The rotation angle in the sagittal plane; *β*, The rotation angle in the coronal plane; *γ*, the rotation angle in the horizontal plane.

*** *p* < 0.001.

**** *p* < 0.0001. Partial eta-squared (*η*^2^): the effect size of factors on independent variables. 0.01: small effect size; 0.06: medium effect size; 0.14: large effect size.

## Discussion

Approximately one-third of patients with dentofacial deformities present with asymmetric deformity (20). We found 122 patients with dentofacial deformity and 30% (36 case) of them had asymmetry. Orthodontic-orthognathic treatment is currently the primary approach to correct asymmetric maxillofacial deformities. However, due to changes in occlusion and jaw movement after surgery, studies suggest that the mandibular condyle may showing remodeling and displacement (21, 22). The extent of condylar remodeling plays a critical role in maintaining postoperative occlusal stability, preventing bony recurrence, and preserving TMJ health (23).

In our earlier study, we developed a reliable and efficient method for assessing condylar changes using deep learning (16), which provides reliable and stable CBCT image analysis. In that study, we focused on the patients with skeletal class II malocclusion and found condylar resorption in both sides after bimaxillary orthognathic surgery, correlated with counterclockwise rotation in the sagittal plane. In the current study, we used a new sample who were diagnosed with asymmetric dentofacial deformity.

We found the average volume of the condyle on the deviated side is smaller than that on the non-deviated side, which is consistent with Chou's findings (24). However, some studies have reported that the deviated condyle is larger than the non-deviated condyle (3, 25, 26). This discrepancy may because of the different marking point of the condylar extent in other studies.

As a result of jaw repositioning and occlusal changes, the condyle may undergo varying degrees of remodeling after orthognathic surgery (21–23, 27). In each side, there were no significant difference in condylar volume between T0T1, suggesting that condylar volume remained stable during the immediate postoperative phase. Between T1T2, we found the deviated side condylar volume decreased, aligning with many studies that condylar remodeling occurs after orthognathic surgery (22). However, we found the non-deviated condylar reduction after surgery was minimal. Many studies indicate that patients with skeletal Class II dentofacial deformities exhibit progressive bilateral condylar resorption and remodeling postoperatively (19, 28). In contrast, the condyle of patients with Class III deformities exhibited greater postoperative stability (29). This phenomenon may result from the anterior movement of the distal mandibular segment (30). Postoperatively, musculofascial tension induces a posterior force, pushing the condyle into the fossa (31). This generates compressive loading on the condylar head, initiating adaptive remodeling. For the deviated side, the movement of the distal mandibular were similar to skeletal Class II patients, thereby resulting in similar outcomes.

Most of the changes observed were very small in magnitude or even practically insignificant, and some of the smaller changes may fall within the error margin. However, even relatively small but true changes in TMJ position could lead to major occlusal problems affecting dental relationships.

Several studies suggest that condyles undergo three-dimensional displacement immediately after orthognathic surgery (32). In the anterior-posterior direction, we had not found significantly difference. However, Baek et al. (33) reported the condyle displaces posteriorly but Kim et al. (34) observed the contrary phenomena. We found condyles shifted outward laterally and downward vertically, in both sides immediately after surgery. Choi et al. (5), Zhang et al. (13) and Svetlana Tyan et al. (35) reported the similar phenomena. And we noticed that the position of condyle gradually returns to its original orientation over the long-term post-surgery. However, we find that the condyle on the deviated side has moved upward beyond its original position, as shown in some studies (36–38, 39). It may be related to the remodeling of the condyle. Numerous studies of patients with Class II deformities have identified a correlation between condylar resorption and upward condylar displacement. Thus, both operation and postoperative orthodontic treatment, should avoid imposing excessive loads on the deviated condyle.

In the rotation change of condyle, either on the deviated side or the non-deviated side, we investigated that the condyles rotated clockwise in the sagittal plane, aligning with findings by Ma et al. (11). In contrast, Hsu et al. (18) reported counterclockwise rotation in the sagittal plane in their cohort whereas other studies (17, 19, 40) documented negligible sagittal-plane rotational changes postoperatively. Notably, excessive counterclockwise mandibular rotation in the sagittal plane during orthognathic procedures has been associated with elevated risks of progressive condylar resorption (30). Many studies demonstrated that the condyle rotates anteriorly in the axial plane postoperatively (11, 15, 18, 19, 41), consistent with our findings. Some scholars have suggested that this phenomenon may be related to the fact that most surgeons are right-handed (15). We found that the mostly the condylar rotation returned to the original direction in the long-term, the deviated side condyle had the trend to back to its original direction in the axial plane.

Notably, we found that the condyle on the deviated side rotated laterally in the coronal plane, while the other side rotated medially. These aligns with some prior research findings (19, 22, 42–44), but some others believe that the condyle has no rotational changes in the coronal direction (42, 45).

Some scholars attribute this to the changes in mandibular body width, which is associated with the mandible's “V”-shaped structure and the movement of the distal bone segment (18). For example, on the non-deviated side, posterior movement of the distal segment causes the outward displacement of the proximal segment, causing medial rotation of the condyle. Podčernina et al. (19) concluded that the bilateral condyles in skeletal Class III patients rotated medially after surgery, resembling the medial rotation on the non-deviated side condyle after the posterior shift of the distal bone segment. However, some studies have reported findings that contradict our results (46, 47).

**Figure 6:**
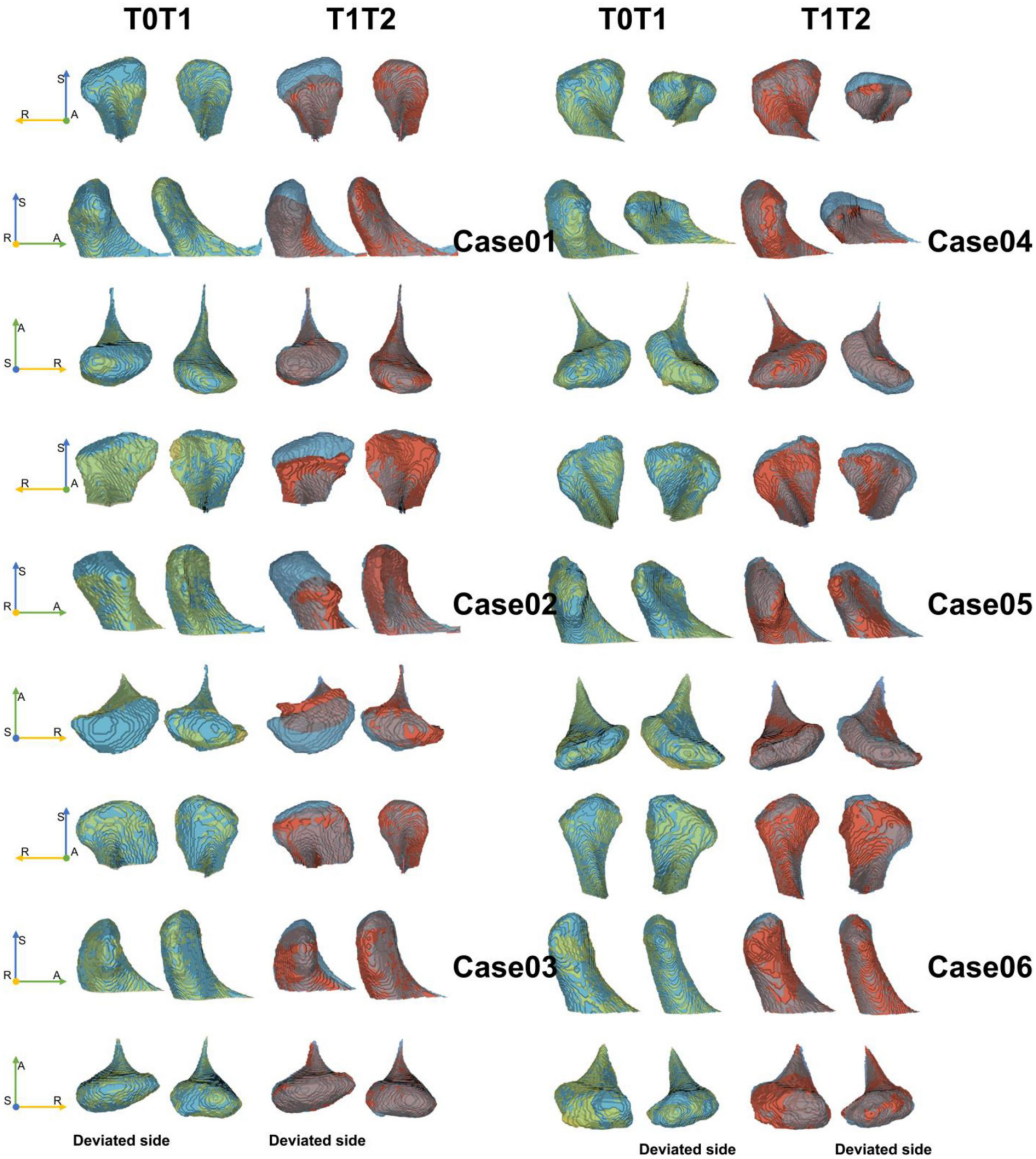
Six cases of condylar morphology changes between T0T1 and T1T2. A, anterior; R, right; S, superior. Yellow area refers to the picture of T0, and blue area refers to the picture at T1, and red area refers to the picture at T2.

Orthognathic surgery not only alters mandibular positioning and occlusal relationships, but also directly impacts the morphology of condyle (Figure 6). Condylar displacement and adaptive remodeling after surgery are critically associated with clinical outcomes (12, 15). Emerging evidence suggests that despite successful restoration of occlusal relationships postoperatively, persistent TMJ instability may predispose patients to TMJ discomforts and relapse (17, 19). Comprehensive evaluation of postoperative temporomandibular joint (TMJ) alterations and predicting the reshaping of condyle after surgery, enables surgeons to develop holistic surgical strategies.

The current study still has some limitations, including the application of artificial intelligence to analyze 3D biomedical images. Due to lack of robust mandibular orientation, it remains insufficiently standardized to support reliable measurement of small changes in the condyles. Another limitation is the lack of reliability testing and more samples from multicenter are needed for further analyze. Moreover, we cannot reveal the relation between condylar changes and TMJ function. Future investigations will incorporate patients with TMJ discomfort to delineate associations between postoperative condylar kinematics and clinical symptomatology, thereby advancing our understanding of biomechanical contributors to TMJ dysfunction in orthognathic outcomes. And it also needs to quantify relationships between mandibular movement magnitude, condylar displacement/rotation, and volumetric remodeling.

## Conclusion

For patients with asymmetric dentofacial deformities, orthognathic surgery causes positional and rotational changes. Condyle in the both sides tend to return to its original position in the long run, but there are still measurable rotations in the coronal plane. Condyle on the deviated side undergo greater remodeling than the non-deviated side.

## Data Availability

The raw data supporting the conclusions of this article will be made available by the authors, without undue reservation.
